# Effects of zoledronic acid versus clodronic acid on skeletal morbidity in patients with newly diagnosed multiple myeloma (MRC Myeloma IX): secondary outcomes from a randomised controlled trial

**DOI:** 10.1016/S1470-2045(11)70157-7

**Published:** 2011-08

**Authors:** Gareth J Morgan, J Anthony Child, Walter M Gregory, Alex J Szubert, Kim Cocks, Sue E Bell, Nuria Navarro-Coy, Mark T Drayson, Roger G Owen, Sylvia Feyler, A John Ashcroft, Fiona M Ross, Jennifer Byrne, Huw Roddie, Claudius Rudin, Gordon Cook, Graham H Jackson, Ping Wu, Faith E Davies, on behalf of the National Cancer Research Institute Haematological Oncology Clinical Studies Group

**Affiliations:** a Institute of Cancer Research, Royal Marsden NHS Foundation Trust, London, UK; b Clinical Trials Research Unit, University of Leeds, Leeds, UK; c University of Birmingham, Birmingham, UK; d St James's University Hospital, Leeds, UK; e Calderdale and Huddersfield NHS Trust, Huddersfield, UK; f Mid Yorkshire Hospitals NHS Trust, Wakefield, UK; g Wessex Regional Genetics Laboratory, University of Southampton, Salisbury, UK; h Nottingham University Hospitals, Nottingham, UK; i Western General Hospital, Edinburgh, UK; j Royal Devon and Exeter Hospital, Exeter, UK; k University of Newcastle, Newcastle-upon-Tyne, UK

## Abstract

**Background:**

Bisphosphonates are the standard of care for reducing the risk of skeletal-related events in patients with bone lesions from multiple myeloma. The MRC Myeloma IX study was designed to compare the effects of zoledronic acid versus clodronic acid in newly diagnosed patients with multiple myeloma. Here, we report the secondary outcomes relating to skeletal events.

**Methods:**

Patients (≥18 years) with newly diagnosed multiple myeloma were enrolled from 120 centres in the UK and received intensive or non-intensive antimyeloma treatment. A computer-generated randomisation sequence was used to allocate patients in a 1:1 ratio, through an automated telephone service to intravenous zoledronic acid (4 mg every 21–28 days) or oral clodronic acid (1600 mg/day), and the drugs were continued at least until disease progression. No investigators, staff, or patients were masked to treatment allocation. The primary endpoints—overall survival, progression-free survival, and overall response rate—and adverse events have been reported previously. We assessed between-group differences with Cox proportional hazards models for time to first skeletal-related event and incidence of skeletal-related events. These were defined as fractures, spinal cord compression, radiation or surgery to bone, and new osteolytic lesions. Data were analysed until disease progression. Analyses were by intention to treat. This trial is registered, number ISRCTN68454111.

**Findings:**

1960 patients were randomly assigned and analysed—981 in the zoledronic acid group and 979 in the clodronic acid group. This trial is fully enrolled, and follow-up continues. At a median follow-up of 3·7 years (IQR 2·9–4·7), patients in the zoledronic acid group had a lower incidence of skeletal-related events than did those in the clodronic acid group (265 [27%] *vs* 346 [35%], respectively; hazard ratio 0·74, 95% CI 0·62–0·87; p=0·0004). Zoledronic acid was also associated with a lower risk of any skeletal-related event in the subsets of patients with (233 [35%] of 668 *vs* 292 [43%] of 682 with clodronic acid; 0·77, 0·65–0·92; p=0·0038) and without bone lesions at baseline (29 [10%] of 302 *vs* 48 [17%] of 276 with clodronic acid; 0·53, 0·33–0·84; p=0·0068). Fewer patients in the zoledronic acid group had vertebral fractures than did those in the clodronic acid group (50 [5%] in the zoledronic acid group *vs* 88 [9%] in the clodronic acid group; p=0·0008), other fractures (45 [5%] *vs* 66 [7%]; p=0·04), and new osteolytic lesions (46 [5%] *vs* 95 [10%]; p<0·0001).

**Interpretation:**

The results of this study support the early use of zoledronic acid rather than clodronic acid in patients with newly diagnosed multiple myeloma for the prevention of skeletal-related events, irrespective of bone disease status at baseline.

**Funding:**

Medical Research Council (London, UK), Novartis, Schering Health Care, Chugai, Pharmion, Celgene, and Ortho Biotech.

## Introduction

Multiple myeloma, which is diagnosed in more than 100 000 people every year,^1^ is characterised by the growth of malignant plasma cells in the bone marrow.^2^, ^3^ Interactions between myeloma cells and bone marrow stromal cells are fundamental to the excessive activation and proliferation of osteoclasts, causing localised bone destruction.^4^ Myeloma cells also secrete factors that inhibit osteoblasts, blocking the repair of osteolytic damage. The resulting bone lesions place patients at risk of skeletal-related events^5^ such as pathological fractures, the need for surgery or palliative radiation to the bone, and spinal cord compression.^6^

Bisphosphonates, such as clodronic acid, pamidronic acid, and zoledronic acid, inhibit osteoclast-mediated osteolysis and are the pharmacological standard of care for patients with myeloma bone disease.^4^, ^7^, ^8^ Clodronic acid reduced skeletal morbidity in an earlier Medical Research Council (MRC) study in patients with multiple myeloma,^9^, ^10^ and as a result has become a widely adopted, standard treatment in the UK.^11^

In the MRC Myeloma IX trial, we investigated whether the addition of an oral (clodronic acid) or an intravenous bisphosphonate (zoledronic acid) to antimyeloma treatment could improve clinical outcomes in patients with newly diagnosed multiple myeloma. Results from this trial showed that patients given zoledronic acid had significantly improved progression-free survival (hazard ratio [HR] 0·88, 95% CI 0·80–0·98; p=0·0179) and a reduced risk of death (0·84, 0·74–0·96; p=0·0118) versus clodronic acid, with overall survival prolonged by 5·5 months.^12^ Importantly, improved overall survival with zoledronic acid remained significant after adjustment for the effect of skeletal-related events (0·85, 0·74–0·97; p=0·018), suggesting that zoledronic acid has direct antimyeloma activity. In this analysis, we investigated in detail the effects of clodronic acid and zoledronic acid on skeletal-related events in patients with newly diagnosed multiple myeloma.

## Methods

### Trial design

The MRC Myeloma IX trial was a multicentre (n=120), randomised, open-label, two-by-two factorial trial, with equal group allocation that was designed to compare primary and maintenance treatments and the effects of an oral (clodronic acid) versus an intravenous bisphosphonate (zoledronic acid). Full details have already been reported.^12^

### Patients

Adult patients (≥18 years) with newly diagnosed and histologically confirmed symptomatic multiple myeloma were eligible for inclusion in the trial. Patients with evidence of bone lesions on axial skeletal survey or fracture at baseline were defined as having myeloma bone disease. Exclusion criteria included previous or concurrent active tumours, acute renal failure (defined as serum creatinine concentration >500 μmol/L that was unresponsive to 72 h of rehydration, urine output <400 mL/day, or requirement for dialysis), and previous treatment for multiple myeloma (except local radiotherapy for bone pain or spinal cord compression, bisphosphonates for hypercalcaemia of malignancy, or low-dose corticosteroids).

The trial was approved by the North West Multi-centre Research Ethics Committee and local review committees at all participating centres. All patients provided written informed consent.

### Randomisation and masking

The methods of randomisation and masking for this study have been previously described in detail.^12^ Briefly, a computer-generated randomisation sequence was used to allocate patients in a 1:1 ratio by use of an automated telephone service to zoledronic acid or clodronic acid. No investigators, staff, or patients were masked to treatment allocation.

### Treatment

Patients were allocated to two main treatment pathways (intensive and non-intensive), as previously described in detail.^12^ In each pathway, patients were randomly assigned to oral clodronic acid (1600 mg/day) or intravenous zoledronic acid (4 mg, 15 min infusion every 3–4 weeks with induction chemotherapy and every 4 weeks thereafter). Dose adjustment for patients with impaired renal function at baseline and delays in administration of the dose in patients with increases in serum creatinine concentration during the study were implemented for zoledronic acid, per the prescribing information. After first-line treatment, eligible patients were randomly assigned to maintenance therapy with thalidomide (50 mg/day initially, increasing to 100 mg/day if tolerated) or no thalidomide maintenance. Bisphosphonates and maintenance therapy were administered continuously at least until disease progression.

Oral health recommendations were provided to investigators from June, 2006, based on the recommendations of Weitzman and colleagues^13^ to reduce the risk of osteonecrosis of the jaw and to identify and manage suspected cases of this adverse event.^13^

### Assessments

The incidence and type of adverse events and serious adverse events were assessed with continuous monitoring. Treatment-emergent serious adverse events were those judged by the treating physician to be related to study drugs. Serum creatinine concentration was monitored every month during induction chemotherapy. After induction chemotherapy, the concentration was monitored every month for patients in the zoledronic acid group and every 3 months for those in the clodronic acid group. Data for skeletal-related events, defined as vertebral fractures, other fractures, spinal cord compression, need for radiation or surgery for bone lesions, and new osteolytic lesions, were analysed until disease progression. A new osteolytic lesion was first recorded as a skeletal-related event, and then as disease progression. Indicators of skeletal-related events were collected every 3 months. These included bone fracture, radiation to bone, surgery for bone lesions and spinal cord compression, and height loss (as an indicator for further imaging follow-up). Dorsal and lumbar spine radiographs were also assessed at baseline and every year after the initial randomisation. Further imaging was initiated in patients who developed clinical symptoms (eg, bone pain) and at disease progression. A specific case-report form was used to report the results of skeletal imaging. Additional prespecified analyses of hypercalcaemia of malignancy and exploratory analyses of skeletal-related events, excluding the development of new osteolytic lesions, were undertaken.

### Statistical analysis

The primary endpoints of progression-free survival, overall survival, and overall response rate, and the secondary endpoint of safety have been reported previously.^12^ Here, we report the secondary endpoint of skeletal-related events. The sample size was calculated based on the comparison of chemotherapy regimens in the factorial design. In the intensive pathway, we aimed to recruit 1080 patients (540 per group) to test the hypothesis that cyclophosphamide, thalidomide, and dexamethasone (CTD) was not inferior to cyclophosphamide, vincristine, doxorubicin, and dexamethasone (CVAD), with a hazard ratio of 1·2 and 80% power at a 5% significance level. In the non-intensive pathway, we aimed to recruit 850 patients (425 per group) to assess whether attenuated CTD (CTDa) was superior to standard chemotherapy with melphalan plus prednisolone, with 80% power at a 5% significance level. We calculated that the sample size for the intensive and non-intensive pathways combined had sufficient power (>80%) to detect a reduction of 10% in the proportion of patients with skeletal-related events for zoledronic acid compared with clodronic acid.

Analyses were based on the treatment that patients with histologically confirmed multiple myeloma who provided written informed consent were randomly assigned to receive (intention-to-treat population). Time to first skeletal-related event (both with and without new osteolytic lesions included as a skeletal-related event) was assessed by use of a cumulative incidence function.^14^ A Cox model for skeletal-related events was generated that included the minimisation factors (centre, haemoglobin, serum calcium, serum creatinine, and platelet count), chemotherapy, and the history of skeletal-related events at baseline (stratified by pathway). Confirmatory, unadjusted analyses (not reported) were also undertaken and provided results similar to the adjusted analyses. Relative risks of all on-study skeletal-related events were assessed with a mean cumulative function. To reduce the potential effects of related skeletal-related events (eg, a fracture requiring surgery) in multiple-event analyses, only one skeletal-related event per 21 days was included (eg, skeletal-related events of possibly linked causality were counted only once, and judged to be one skeletal-related event). Post-hoc analyses that included all skeletal-related events irrespective of whether they were the first or subsequent skeletal-related events within 21 days (not reported) were done and provided results consistent with those in which the linked events were counted as one skeletal-related event. A Cox model for all on-study skeletal-related events was generated that included the same variables as the Cox model for time to first skeletal-related events.^15^ Statistical analysis was done with SAS (version 9.2) and Digital Visual Fortran software (version 6.0A). All hypothesis tests were two-sided and undertaken at the 5% significance level, without adjustment for multiplicity.

This trial is registered, number ISRCTN68454111.

### Role of the funding source

No funding organisation was involved in study design, data collection, data analysis or interpretation, writing, or decision about publication submission. All authors had full access to trial data; GJM, JAC, and GHJ had final responsibility for the decision to submit for publication.

## Results

**Figure 1:**
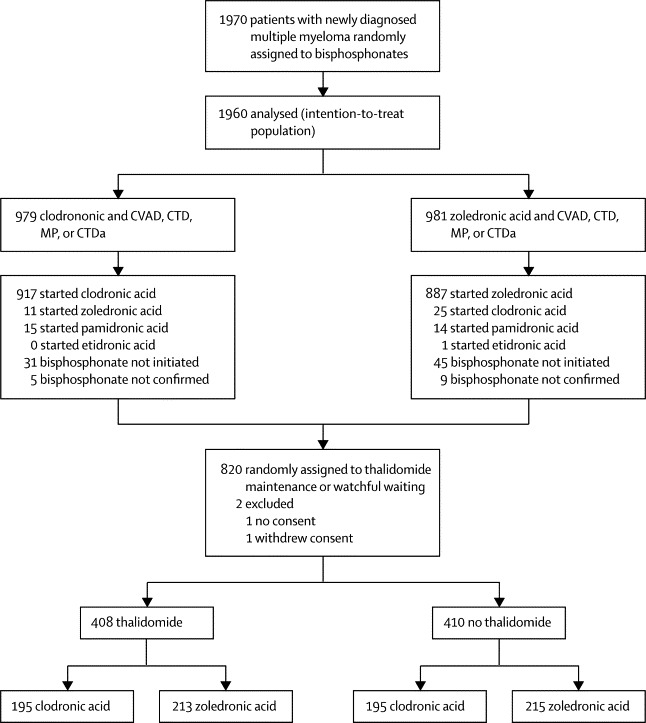
Trial profile The complete trial protocol is provided in Morgan and colleagues.^12^ CVAD=cyclophosphamide, vincristine, doxorubicin, and dexamethasone. CTD=cyclophosphamide, thalidomide, and dexamethasone. MP=melphalan and prednisolone. CTDa=attenuated CTD.

1970 patients were enrolled between May 14, 2003, and Nov 20, 2007, and 1960 were the intention-to-treat population (figure 1). Baseline demographics and disease characteristics of patients were well balanced between the zoledronic acid and clodronic acid groups (table 1).^12^ 1898 (97%) of 1960 patients were white, 1401 (71%) had myeloma bone disease at baseline, 562 (29%) had a history of vertebral fractures, 231 (12%) had previous non-vertebral fractures, 1010 (52%) had been diagnosed with osteolytic lesions, and 258 (13%) had previous radiotherapy (table 1). Median follow-up was 3·7 years (IQR 2·8–4·7) for patients in the zoledronic acid group and 3·8 years (2·9–4·7) for those in the clodronic acid group; 582 (30%) patients had been given zoledronic acid or clodronic acid for at least 2 years (290 [30%] of 981 in the zoledronic acid group and 292 [30%] of 979 in the clodronic acid group).

**Table 1:** Baseline demographics and disease characteristics of the intention-to-treat population

		**Intensive pathway**		**Non-intensive pathway**	
		Zoledronic acid (n=555)	Clodronic acid (n=556)	Zoledronic acid(n=426)	Clodronic acid (n=423)
Age (years; median, range)		59 (31–74)	59 (33–78)	73 (59–89)	73 (57–88)
Sex					
	Female	201 (36%)	218 (39%)	191 (45%)	185 (44%)
	Male	354 (64%)	338 (61%)	235 (55%)	238 (56%)
Ethnic origin					
	White	537 (97%)	534 (96%)	412 (97%)	415 (98%)
	Black African	2 (<1%)	8 (1%)	2 (<1%)	2 (<1%)
	Black Caribbean	2 (<1%)	5 (<1%)	6 (1%)	1 (<1%)
	Chinese	7 (1%)	6 (1%)	1 (<1%)	2 (<1%)
	Polynesian	1 (<1%)	0	0	0
	Other	6 (1%)	1 (<1%)	3 (<1%)	1 (<1%)
	Data not available	0	2 (<1%)	2 (<1%)	2 (<1%)
International Staging System stage					
	I	129 (23%)	146 (26%)	63 (15%)	47 (11%)
	II	198 (36%)	182 (33%)	139 (33%)	173 (41%)
	III	174 (31%)	169 (30%)	173 (41%)	160 (38%)
	Data not available	54 (10%)	59 (11%)	51 (12%)	43 (10%)
Hyperdiploidy					
	Yes	159 (29%)	171 (31%)	117 (27%)	132 (31%)
	No	133 (24%)	132 (24%)	87 (20%)	76 (18%)
	Data not available	263 (47%)	253 (46%)	222 (52%)	215 (51%)
Bone disease					
	Yes	404 (73%)	411 (74%)	291 (68%)	295 (70%)
	No	149 (27%)	138 (25%)	130 (31%)	123 (29%)
	Data not available	2 (<1%)	7 (1%)	5 (1%)	5 (1%)
Bone disease or other skeletal-related event					
	Yes	393 (71%)	406 (73%)	275 (65%)	276 (65%)
	No	159 (29%)	141 (25%)	143 (34%)	135 (32%)
	Data not available	3 (<1%)	9 (2%)	8 (2%)	12 (3%)
Bone pain					
	Yes	428 (77%)	415 (75%)	275 (65%)	287 (68%)
	No	120 (22%)	132 (24%)	147 (35%)	131 (31%)
	Data not available	7 (1%)	9 (2%)	4 (<1%)	5 (1%)
Baseline radiotherapy to bone					
	Yes	80 (14%)	86 (15%)	43 (10%)	49 (12%)
	No	473 (85%)	469 (84%)	382 (90%)	372 (88%)
	Data not available	2 (<1%)	1 (<1%)	1 (<1%)	2 (<1%)
Baseline vertebral fractures					
	Yes	152 (27%)	166 (30%)	114 (27%)	130 (31%)
	No	388 (70%)	373 (67%)	302 (71%)	286 (68%)
	Data not available	15 (3%)	17 (3%)	10 (2%)	7 (2%)
Other baseline fractures					
	Yes	74 (13%)	73 (13%)	42 (10%)	42 (10%)
	No	460 (83%)	460 (83%)	376 (88%)	371 (88%)
	Data not available	21 (4%)	23 (4%)	8 (2%)	10 (2%)
Baseline osteolytic lesions					
	Yes	303 (55%)	293 (53%)	209 (49%)	205 (48%)
	No	239 (43%)	247 (44%)	212 (50%)	207 (49%)
	Data not available	13 (2%)	16 (3%)	5 (1%)	11 (3%)
Calcium after hydration (mmol/L)					
	Median (IQR)	2·4 (2·2–2·5)	2·4 (2·3–2·5)	2·4 (2·2–2·5)	2·4 (2·3–2·5)
	Data not available	37 (7%)	51 (9%)	37 (9%)	37 (9%)

Data are number (%), unless otherwise indicated. Data, in part, from Morgan and colleagues.^12^

**Figure 2:**
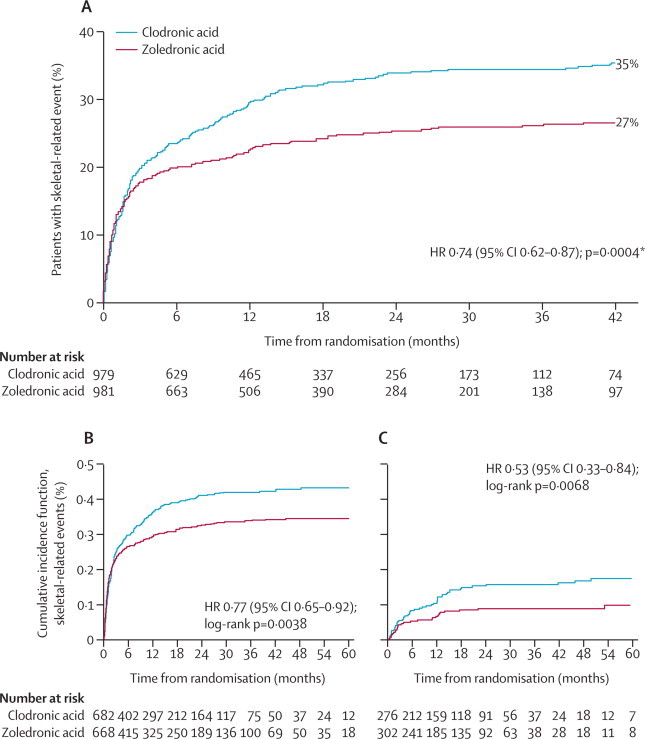
Time to first skeletal-related event overall (A), in patients with bone lesions at baseline (B), and in patients without bone lesions at baseline (C) HR=hazard ratio. *Cox p value.

Overall, 611 patients had a skeletal-related event before or as the first event of disease progression (eg, new osteolytic lesion) during the study. In the overall patient population, fewer patients assigned to zoledronic acid had a skeletal-related event than did those assigned to clodronic acid (265 [27%] of 981 *vs* 346 [35%] of 979, respectively), with zoledronic acid significantly reducing the risk of skeletal-related events versus clodronic acid (figure 2A). Unadjusted analyses provided similar results (data not shown). The total number of skeletal-related events reported was also lower in the zoledronic acid group than in the clodronic acid group (419 *vs* 597, respectively). The proportion of patients with at least one skeletal-related event was consistently lower in the zoledronic acid group versus the clodronic acid group at each timepoint (p<0·0001 overall; table 2). The difference in incidences of skeletal-related events for zoledronic acid versus clodronic acid was significant for each of the first 3 years separately, but the total number of skeletal-related events was low at 48 months and 60 months, and reduced the statistical power for the respective 12-month comparisons (table 2). Similar distributions of skeletal-related events were noted for patients treated with zoledronic acid and clodronic acid in the intensive (zoledronic acid, 155 [28%] of 555; clodronic acid, 202 [36%] of 556; log-rank p=0·003) and non-intensive pathways (zoledronic acid, 110 [26%] of 426; clodronic acid, 144 [34%] of 423; log-rank p=0·008). The mean skeletal morbidity rate was lower with zoledronic acid versus clodronic acid in the intensive and non-intensive pathways (0·4 skeletal-related events per patient per year *vs* 0·8 per patient per year, respectively).

**Table 2:** Cumulative annual incidence of first and subsequent skeletal-related events for the intention-to-treat population after randomisation

	**Skeletal-related events**		**Incidence (95% CI)**		Overall difference (95% CI) between clodronic acid and zoledronic acid*	**p value for preceding 12 months**†
	Clodronic acid (n=979)	Zoledronic acid (n=981)	Clodronic acid (n=979)	Zoledronic acid (n=981)		
12 months	451 (46%)	333 (34%)	0·43 (0·38–0·48)	0·33 (0·28–0·37)	0·11 (0·04–0·18)	0·0002
24 months	93 (9%)	54 (6%)	0·60 (0·53–0·66)	0·42 (0·36–0·48)	0·18 (0·09–0·26)	0·0024
36 months	28 (3%)	16 (2%)	0·69 (0·61–0·78)	0·47 (0·40–0·53)	0·23 (0·12–0·33)	0·0089
48 months	13 (1%)	4 (<1%)	0·80 (0·68–0·91)	0·49 (0·42–0·56)	0·30 (0·17–0·44)	0·0510
60 months	2 (<1%)	2 (<1%)	0·83 (0·70–0·95)	0·51 (0·43–0·58)	0·32 (0·18–0·46)	0·5359

Data are number (%), unless otherwise indicated.

* p<0·0001.

† Unadjusted p value for the comparison of incidence of skeletal-related events in zoledronic acid group versus clodronic acid group per 12 months (eg, 24-month p value is for incidence between 12 months and 24 months).

At enrolment, all patients had histologically confirmed symptomatic multiple myeloma. However, patients who did not have myeloma bone disease at baseline had lower rates of bone pain (248 [43%] of 578 *vs* 1136 [84%] of 1350), and higher rates of anaemia (330 [57%] *vs* 523 [39%]), renal failure (96 [17%] *vs* 171 [13%]), and infection (74 [13%] *vs* 90 [7%]) than did patients with bone disease at baseline.

In an exploratory analysis that excluded new osteolytic lesions from the definition of skeletal-related event, the outcome was similar to the analysis that included new osteolytic lesions, and the reduction in risk of skeletal-related events with zoledronic acid was still significant (HR 0·76, 95% CI 0·64–0·89; log-rank p=0·0011). Further exploratory analysis by patients' risk of skeletal-related events, as identified in another assessment of the Myeloma IX patients,^16^ showed a reduced risk of skeletal-related events with zoledronic acid versus clodronic acid in the high-risk and low-risk populations (data not shown). Analysis by cytogenetic markers (poor prognosis defined by use of fluorescence in-situ hybridisation [FISH] as adverse IgH translocations, gain of 1q, and loss of 17p) showed that the benefit associated with zoledronic acid in terms of skeletal-related events was attributable to the non-poor prognosis subset,^16^ wherein the benefits of zoledronic acid were especially meaningful (log-rank p=0·0012; data not shown). In the poor-prognosis subset, disease progression was fairly rapid, and very few patients were assessable for the time to first skeletal-related-event endpoint at later timepoints (48 months and 60 months; data not shown).

**Figure 3:**
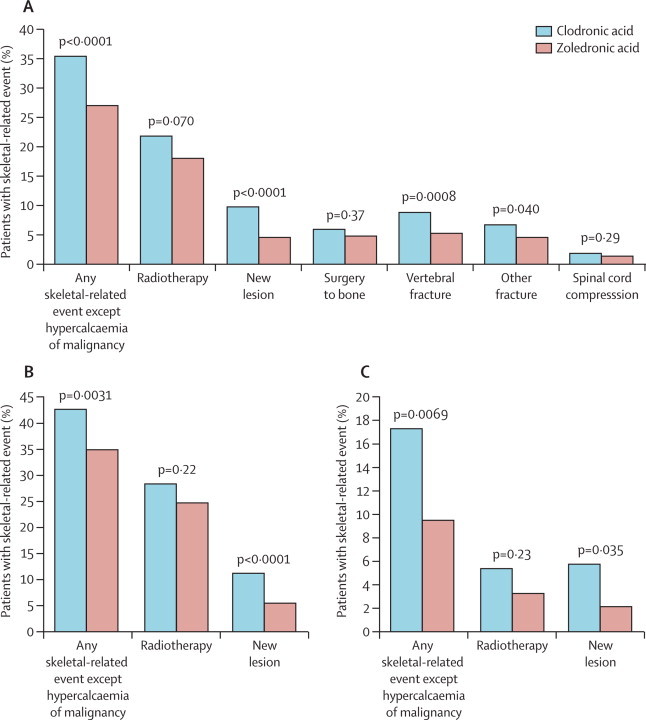
Proportion of patients with an on-study skeletal-related event overall (A), with bone lesions at baseline (B), and without bone lesions at baseline (C)

In an exploratory analysis, patients with osteolytic lesions at baseline showed a non-significantly longer progression-free survival than did those with no osteolytic lesions (20 months, 95% CI 18–21, *vs* 18 months, 17–19, HR 0·90, 0·82–1·00; p=0·0535). However, patients with bone disease at baseline had a higher incidence of skeletal-related events than did those without bone disease at baseline (525 [39%] of 1350 *vs* 77 [13%] of 578, respectively; p<0·0001). Moreover, zoledronic acid, compared with clodronic acid, was associated with a significantly reduced risk of any skeletal-related event in patients with (233 [35%] of 668 *vs* 292 [43%] of 682; figure 2B) and without bone disease at baseline (29 [10%] of 302 *vs* 48 [17%] of 276; figure 2C). Zoledronic acid was associated with a reduced incidence of each type of skeletal-related event versus clodronic acid in the overall population (figure 3A), and significant reductions were noted for any skeletal-related event (265 [27%] of 981 *vs* 346 [35%] of 979 for clodronic acid; p<0·0001), new osteolytic lesions (46 [5%] *vs* 95 [10%] for clodronic acid; p<0·0001), vertebral fractures (50 [5%] *vs* 88 [9%] for clodronic acid; p=0·0008), and other fractures (45 [5%] *vs* 66 [7%] for clodronic acid; p=0·04), but not radiotherapy (179 [18%] *vs* 211 [22%] for clodronic acid; p=0·07), surgery to the bone (49 [5%] *vs* 58 [6%] for clodronic acid; p=0·37), and spinal-cord compression (13 [1%] *vs* 19 [2%] for clodronic acid; p=0·29). Further details about the other fracture sites were not collected during the study. Zoledronic acid was associated with a significantly reduced incidence of any skeletal-related event in patients with (figure 3B) and without bone disease at baseline (figure 3C).

In a prespecified multivariate model of all on-study skeletal-related events, the reduction in such events with zoledronic acid versus clodronic acid remained significant (table 3). Baseline serum calcium concentration, baseline skeletal-related event, melphalan plus prednisolone versus CTDa (in the non-intensive pathway), and treatment centre also showed significant correlations with risk of skeletal-related events (table 3). Overall, exclusion of new osteolytic lesions from the skeletal-related-events composite endpoint did not affect model outcomes (data not shown).

**Table 3:** Multivariate model for risk of skeletal-related events

		**Hazard ratio (95% CI)**	**p value**
Zoledronic acid *vs* clodronic acid		0·72 (0·62–0·84)	<0·0001
CTD *vs* CVAD (intensive pathway)		0·91 (0·76–1·10)	0·3399
CTDa *vs* melphalan+prednisolone (non-intensive pathway)		0·75 (0·60–0·94)	0·0141
Serum calcium concentration (high *vs* low)		1·31 (1·11–1·55)	0·0012
Serum creatinine concentration (high *vs* low)		0·93 (0·77–1·12)	0·4362
Haemoglobin concentration (high *vs* low)		1·08 (0·92–1·26)	0·3489
Platelets (high *vs* low)		1·19 (0·93–1·53)	0·1623
Skeletal-related events at baseline*			
	No *vs* yes	0·35 (0·28–0·44)	<0·0001
	Missing *vs* yes	0·83 (0·46–1·52)	0·5532
Centre†		..	<0·0001

CTD=cyclophosphamide, thalidomide, and dexamethasone. CVAD=cyclophosphamide, vincristine, doxorubicin, and dexamethasone. CTDa=attenuated CTD.

* Any event, including diagnosis of a osteolytic lesion.

† Overall p value, but not hazard ratio, reported for 120 centres.

Results of further exploratory analyses showed that the proportion of patients with a skeletal-related event was significantly lower with zoledronic acid versus clodronic acid when the first 12 months (log-rank p=0·0012) or 24 months (log-rank p=0·0102) were excluded from the analyses (data not shown). Furthermore, significantly fewer patients in the zoledronic acid group than in the clodronic acid group had at least one skeletal-related event after randomisation to maintenance thalidomide or no maintenance (log-rank p=0·0005; data not shown).

The incidences of most adverse events were similar in zoledronic acid and clodronic acid groups and have been previously reported.^12^ Overall, the rates of acute renal failure were low and similar for patients in the zoledronic acid and clodronic acid groups (57 [6%] of 983 [two patients for whom confirmation of consent was not received were included in the safety population] *vs* 60 [6%] of 979, respectively; p=0·78). During the study, 47 (5%) of 979 patients in the clodronic acid group and 43 (4%) of 981 in the zoledronic acid group died of renal failure (p=0·67), and 123 (13%) patients in the clodronic acid group and 92 (9%) in the zoledronic acid group died of multiple myeloma or treatment-related infections (p=0·025). 28 (3%) patients in the clodronic acid group and 28 (3%) in the zoledronic acid group had hypercalcaemia, which was reported as a serious adverse event in six (<1%) of 979 patients in the clodronic acid group and six (<1%) of 983 in the zoledronic acid group. As previously reported,^12^ confirmed osteonecrosis of the jaw was rare, but the rate was higher in the zoledronic acid group than in the clodronic acid group (35 [4%] *vs* three [<1%], respectively; p<0·0001). Gastrointestinal serious adverse events were not significantly different with clodronic acid versus zoledronic acid (30 [3%] *vs* 24 [2%], respectively; p=0·41). Acute-phase reactions generally were not severe and did not interfere with antimyeloma treatments (data not shown).

Treatment-emergent serious adverse events that were suspected to be related to bisphosphonate use arose in 41 (45 events) of 983 patients in the zoledronic acid group versus 33 (34 events) of 979 patients in the clodronic acid group, and represented a subset of the overall treatment-emergent serious adverse events that were suspected to be related to any of the study drugs as previously reported.^12^ In patients given zoledronic acid, treatment-emergent serious adverse events were musculoskeletal, connective tissue, and bone disorders (n=16); renal and urinary disorders (n=8); haematological disorders (n=5); gastrointestinal disorders (dehydration [n=1], nausea and vomiting, epigastric tenderness [n=1], and nausea, vomiting, constipation, dehydration [n=1]); endocrine, metabolism, or nutrition disorders (n=3); infections (n=3); cardiovascular disorders (n=2); skin and subcutaneous disorders (n=2); fluid or electrolyte disturbance (n=1); general disorder or administration-site condition (n=1); and nervous system disorder (n=1). In patients in the clodronic acid group, treatment-emergent serious adverse events were gastrointestinal disorders (diarrhoea [n=2], abdominal pain and bloating [n=1], nausea or vomiting [n=6], oesophagitis [n=1], haematemesis [n=1], and gastrointestinal disturbance [n=1]); renal and urinary disorders (n=9); infections (n=5); haematological disorders (n=2); skin and subcutaneous disorders (n=2); cardiovascular disorder (n=1); general disorder or administration-site condition (n=1); hepatic disorder (n=1); and reproductive system or breast disorder (n=1).

## Discussion

The results of the current analysis of the MRC Myeloma IX trial show that zoledronic acid was associated with a significantly reduced risk of skeletal-related events versus clodronic acid in patients with newly diagnosed multiple myeloma irrespective of their bone disease status at baseline. Additionally, zoledronic acid was associated with a reduced incidence of skeletal-related events in the intensive and non-intensive pathways, suggesting that all patients undergoing initial treatment for multiple myeloma could benefit from early use of zoledronic acid. Previous analyses showed that zoledronic acid was associated with a significant reduction in the risk of death (HR 0·84, 95% CI 0·74–0·96; p=0·0118) and a significant improvement in progression-free survival (0·88, 0·80–0·98; p=0·0179), providing significant clinical benefits and not just reduction in rates of skeletal-related events (panel).^12^ Although clinical guidelines recommend bisphosphonates for patients with documented bone lesions,^4^, ^7^, ^8^, ^17^ all patients (ie, with or without bone disease at baseline) could benefit when bisphosphonates are begun early in the course of multiple myeloma. Moreover, the reductions in skeletal-related events with zoledronic acid versus clodronic acid noted throughout the course of the trial support the continued use of zoledronic acid in patients with multiple myeloma at least until disease progression, when patients went off study in this trial and data for skeletal-related events were no longer collected. However, the optimum duration of zoledronic acid is not known, and some patients might benefit from continuing zoledronic acid, possibly at a reduced dose, during disease remission. Additional clinical trials are needed to further refine these aspects of treatment.

Panel **Research in context**

**Systematic review**

In addition to the experience obtained from previous MRC myeloma trials of clodronic acid, a review of the reports of clinical trials of a wide variety of combination chemotherapy regimens and bisphosphonates was undertaken and used to develop the 2×2 factorial trial design for the Myeloma IX trial. At the time that the Myeloma IX trial was initiated, most patients with symptomatic multiple myeloma in the UK were treated long-term with bisphosphonates; however, there was no consensus for the optimum timing and duration of treatment with bisphosphonates, or the efficacy for prevention of skeletal-related events of all the available bisphosphonates and their potential to affect disease outcomes and survival variables in patients with multiple myeloma. In the Myeloma IX trial, we assessed the possible enhanced effects on disease-related bone changes and survival of a third-generation bisphosphonate (zoledronic acid) in comparison with a standard older-generation oral agent (clodronic acid).

**Interpretation**

The results of the Myeloma IX trial showed significant benefits with zoledronic acid versus clodronic acid on progression-free survival, overall survival, and several variables for skeletal-related events, and, to our knowledge, for the first time established the superiority of one bisphosphonate over another in patients with multiple myeloma. Moreover, exploratory analyses have shown significant benefits with treatment before the onset of bone lesions and also with long-term treatment (eg, during maintenance therapy or long-term follow-up). The data from Myeloma IX provide compelling evidence that zoledronic acid should be considered an essential component of therapeutic regimens for patients starting treatment for newly diagnosed multiple myeloma irrespective of whether bone lesions are already present, and support the continuation of zoledronic acid at least until disease progression. However, the results of this study do not provide insight into whether the frequency of dosing with zoledronic acid can be reduced after initial treatment, such as in patients with long-term remission of their disease.

In patients with multiple myeloma, renal adverse events and osteonecrosis of the jaw are causes for concern and should be monitored and managed appropriately. In the Myeloma IX trial, adverse events were consistent with the established tolerability profiles of zoledronic acid and clodronic acid in patients with multiple myeloma. Overall, renal adverse events occurred at a similar rate for both bisphosphonates although clodronic acid is an oral bisphosphonate and not typically associated with renal toxicity. Indeed, because monitoring the concentrations of creatinine in the serum was less frequent for the clodronic acid group than for the zoledronic acid group (ie, every 3 months *vs* monthly, respectively), any observation bias would be in favour of clodronic acid. This bias suggests that the renal adverse events reported in the Myeloma IX trial were most likely a result of the disease, antimyeloma treatments, non-myeloma-related drugs, or other comorbidities rather than toxicity associated with bisphosphonates, validating the renal safety protocols for zoledronic acid. Although osteonecrosis of the jaw arose at a higher rate in patients in the zoledronic acid group, most events were low grade (as defined by Weitzman and colleagues^13^) and manageable. Recommendations for prevention and management of osteonecrosis of the jaw were implemented in Myeloma IX from June, 2006, onwards,^13^ but further reductions in the risk of osteonecrosis of the jaw might be possible with prevention strategies that were reported subsequently. Recent evidence suggests that the risk of osteonecrosis of the jaw can be significantly reduced by the implementation of preventative dental care, most cases are manageable, and complete healing of lesions of osteonecrosis of the jaw is possible.^18^ Although a small percentage of patients in the zoledronic acid group were reported to have adverse events that were consistent with acute-phase reactions (ie, fever unattributed to infection, myalgia, and arthralgia), these did not lead to discontinuation or delay in antimyeloma treatments. The results of a study in postmenopausal women (n=7765) given intravenous zoledronic acid (5 mg per year) for osteoporosis showed that the acute-phase reaction lasted about 3 days and the incidence was similar to that with placebo by the third infusion.^19^ In Myeloma IX, acute-phase reactions, which are thought to result from immune-cell activation after the first infusion of zoledronic acid, might have been less frequent than in the postmenopausal osteoporosis setting because of the fairly high prevalence of disease and treatment-related myelosuppression in patients with multiple myeloma. Gastrointestinal adverse events are generally more common in patients given oral bisphosphonates, as noted here—gastrointestinal treatment-emergent serious adverse events arose more frequently with clodronic acid than with zoledronic acid (12 *vs* three events, respectively).

Skeletal-related events are serious, potentially debilitating complications affecting most patients with multiple myeloma, especially those who do not receive bone-targeted therapy.^6^ Bisphosphonates can effectively reduce the risk of skeletal-related events in this population.^20^, ^21^, ^22^ Consequently, clinical practice guidelines and recommendations include bisphosphonates as a key component of disease treatment for patients with myeloma bone disease.^4^, ^7^, ^8^, ^17^ In the previous MRC Myeloma studies,^9^, ^10^ clodronic acid was of particular benefit (ie, slowing progression of skeletal disease and reducing skeletal morbidity) to patients without bone lesions at treatment initiation.^9^, ^10^ Moreover, subgroup analysis suggested that clodronic acid might confer survival benefits in patients without overt bone disease at diagnosis.^10^

Three bisphosphonates have been approved for patients with osteolytic bone lesions from multiple myeloma—zoledronic acid and pamidronic acid in the USA and Europe, and clodronic acid in Europe. Previous comparison of zoledronic acid with pamidronic acid in patients with multiple myeloma showed no significant difference in the incidence of skeletal-related events (86 [47%] of 183 *vs* 82 [49%] of 167, respectively),^20^ or the time to first skeletal-related event (380 days *vs* 286 days, respectively; p=0·538) among patients with multiple myeloma and established osteolytic lesions.^21^ The more recently introduced nitrogen-containing bisphosphonates, pamidronic acid and zoledronic acid, are more potent inhibitors of osteoclastic activity than are the earlier, non-nitrogen-containing bisphosphonates (eg, clodronic acid).^23^ However, pamidronic acid is more similar to clodronic acid in terms of antiresorptive potency than it is to zoledronic acid, which has more potent anticancer effects in preclinical models of myeloma and other cancers.^23^, ^24^, ^25^ Because of the short infusion time and particular antiresorptive potency of zoledronic acid compared with pamidronic acid—confirmed in preclinical and clinical investigations—it was adopted as the comparator for clodronic acid, a UK standard, in the Myeloma IX Trial. Whether pamidronic acid would have provided similar reductions in skeletal-related events versus clodronic acid in this patient population is not known. Treatment regimens for multiple myeloma also changed between the completion of the trial of zoledronic acid versus pamidronic acid,^21^ and the start of the Myeloma IX trial.

Induction chemotherapy regimens used to treat patients with multiple myeloma are continually evolving as new drugs and new combinations are assessed in clinical trials. For example, the antimyeloma regimens used in the Myeloma IX trial included drugs that are now being combined with newer agents such as bortezomib. In recent years, there has been interest in the potential for immunomodulatory drugs (eg, lenalidomide) and bortezomib to slow osteolytic bone destruction in multiple myeloma through inhibition of osteoclast activity, and increased osteoblast activity (eg, bortezomib).^26^, ^27^, ^28^, ^29^, ^30^ So far, these data are derived from preclinical studies and clinical assessments of biochemical markers of bone resorption and bone mineral density and have not yet been correlated with a reduction in the risk of skeletal-related events. There is no evidence to suggest that antimyeloma treatment alone can replace bisphosphonates for treatment of multiple myeloma bone disease; however, the potential for synergistic effects between these drugs and bisphosphonates is intriguing. On the basis of the data for skeletal-related events obtained in this study, zoledronic acid is very likely to provide clinically significant benefits in combination with newer antimyeloma regimens because it has proved beneficial in the prevention of skeletal-related events in patients with solid tumours who were given a broad range of primary anticancer treatments.^21^, ^31^, ^32^ However, clinical studies are needed to confirm this theory.

The Myeloma IX study is the first large, independent clinical trial in patients with newly diagnosed multiple myeloma, and its results show unequivocal superiority of one bisphosphonate over another for reduction of the risk of skeletal-related events. Overall, the data from this study support the early use of zoledronic acid for reduction in the risk of skeletal-related events in all patients with newly diagnosed multiple myeloma.
